# A study of concurrent chemoradiotherapy with weekly docetaxel and cisplatin for advanced esophageal squamous cell carcinoma with T4 and/or M1 lymph node metastasis or locoregional recurrence

**DOI:** 10.1186/s13014-020-01518-2

**Published:** 2020-04-08

**Authors:** Qi Liu, Yi Xia, Yun Chen, Junhua Zhang, Jiaying Deng, Kuaile Zhao

**Affiliations:** 1 Department of Radiation Oncology, Fudan University Shanghai Cancer Center, 270 Dong’an Road, Shanghai, 200032 China; 2 Department of Oncology, Shanghai Medical College, Fudan University, Shanghai, China; 3 Department of Radiation Oncology, Fudan University Shanghai Cancer Center Minhang Branch Hospital, Shanghai, China

**Keywords:** Esophageal carcinoma, Squamous cell carcinoma, Docetaxel, Cisplatin, Chemoradiotherapy

## Abstract

**Background:**

The improvement of survival outcomes and the reduction of toxicities for esophageal squamous cell carcinoma (SCC) are still needed. We conducted a pilot study of concurrent chemoradiotherapy with weekly docetaxel and cisplatin for the treatment of esophageal SCC with T4 and/or M1 lymph node metastasis (LNM) or locoregional recurrence.

**Methods:**

Fifty-four patients with advanced thoracic esophageal SCC having a stage T4 tumor or M1 LNM and/or locoregional recurrence were enrolled. Docetaxel and cisplatin were both administered weekly at a dose of 25 mg/m^2^ 5–6 times in total concurrently with a specific dose of radiation. The primary endpoint was overall survival (OS), and the secondary endpoints were progression-free survival (PFS), locoregional control and treatment-related toxicities.

**Results:**

From October 2015 to December 2016, concurrent treatment with full-cycle docetaxel and cisplatin and radiotherapy was administered to 41 of 54 patients (75.9%). A total of 51 patients (94.4%) completed the radiation schedules. Twenty-one patients (44.4%) achieved a complete response, and 21 (44.4%) achieved a partial response after chemoradiotherapy. The median survival time was 18.2 months, and the median PFS time was 11.5 months. The 1-year and 3-year OS, locoregional control and PFS rates were 70.4, 80.6, 50.0 and 36.4%, 64.3, 31.5%, respectively. Grade 3 toxicities included neutropenia (13.0%), anemia (3.7%), thrombocytopenia (1.9%), fatigue (20.4%), anorexia (13.0%), esophagitis (11.1%), and pneumonitis (5.6%). Grade 4 neutropenia occurred in 16.7% of patients. Four patients (7.4%) died from grade 5 toxicities. There were no significant differences in both survival and grade 3 and higher toxicities between the newly diagnosed group and recurrent group.

**Conclusions:**

Concurrent chemoradiotherapy with weekly docetaxel and cisplatin is a well-tolerated and effective treatment regimen for esophageal SCC with T4 or M1 LNM and/or locoregional recurrence. Clinical trials with larger sample size and comparisons with conventional fluorouracil and cisplatin regimens are needed.

## Introduction

Esophageal cancer is the fourth most common cause of cancer-related death in China [1]. The main reasons for this are the high rates of local recurrence and metastasis. In Eastern countries, squamous cell carcinoma (SCC) is still the predominant histological subtype of esophageal cancer [2], and a considerable number of patients have already lost the opportunity for treatment with surgery at the time of diagnosis. For patients with nonsurgical esophageal cancer, definitive concurrent chemoradiotherapy (CRT) is the standard treatment, and combined chemotherapy with 5- fluorouracil and cisplatin (PF) is the most commonly used regimen [3–5]. Nevertheless, the outcome of CRT is still unsatisfactory (5-year survival is only 27% according to RTOG 85–01) [4], and 40% of patients treated with concomitant CRT suffer tumor persistence or locoregional recurrence [6]. New active therapies and treatment approaches for esophageal cancer are needed.

Experimental data show that taxanes are particularly effective in less radiation-sensitive squamous cell carcinomas. However, in our recent clinical trial, we did not observe the superiority of paclitaxel over conventional regimens of PF for locally advanced esophageal SCC [7]. The regimen of paclitaxel and carboplatin (TC), which has been a standard regimen of neoadjuvant CRT for esophageal cancer [8], also has an increasing use in definitive CRT. Previous prospective and multi-center retrospective studies found the median overall survival (OS) was 12–13.8 months for inoperable patients who received concurrent CRT with TC regimen [9–11]. Compared with paclitaxel, docetaxel is more active as a promoter of tubulin polymerization and an inhibitor of cell replication in vitro [12]. Docetaxel is a potent radiosensitizer that promotes microtubule stability, causing arrest in the G2 and M phases of the cell cycle and thereby increasing sensitivity to radiation [13]. Cisplatin is probably the most widely used anticancer agent in combination with radiation. Biological evidence suggests that cisplatin causes the inhibition of the repair of radiation-induced DNA damage via both homologous recombination and nonhomologous end-joining. Previous studies have reported encouraging results for the use of a combination of docetaxel and cisplatin (DP) for esophageal cancer concurrently with radiotherapy (RT) [14–17].

System chemotherapy is the standard treatment for those unresectable locally advanced, locally recurrent or metastatic esophageal SCC. However, Ohtsu et al. [18] reported the efficacy of combined radiotherapy with PF for T4 and/or M1 lymph node metastasis (LNM) of squamous cell carcinoma according to the TNM classification scale published by the UICC International Union Against Cancer, 6th edition. Furthermore, the RT dose they used was far from definitive level. Based on our clinical experience, these patients could also benefit from definitive CRT. In a previous study which enrolled only nine patients with advanced thoracic esophageal SCC that had a T4 tumor and/or distant M1 LNM, the response rate was 67%, and the median survival time was 16.2 months with a 2-year survival rate of 38.9% after concurrent treatment with docetaxel weekly and 60 Gy radiation [19].

To gain insight into the relative efficacy and toxicities of DP regimens combined with RT in patients with a T4 tumor and/or distant M1 LNM or locoregional recurrences, we conducted a pilot study of concurrent CRT with a DP regimen for patients diagnosed with locally advanced or recurrent esophageal SCC at our center.

## Materials and methods

### Patients

The criteria for inclusion were: (1) 18–75 years of age; (2) histologically verified squamous cell carcinoma; (3) SCC that was considered unsuitable for surgical resection based on a multidisciplinary team opinion (due to T4 or M1 LNM metastasis, according to TNM classification of the UICC International Union Against Cancer, 6th edition, or patient who had undergone surgery before); (4) ECOG performance status of 0 or 1; (5) adequate organ function to ensure the safety of treatment; (6) life expectancy more than 6 months; (7) use of adequate contraception.

The patients with the following were ineligible: (1) resectable esophageal carcinoma; (2) other active synchronous carcinoma or concurrent uncontrolled medical illness; (3) medical comorbidities that would compromise the delivery of therapy or be exacerbated by the planned treatment; (4) receiving treatment with another investigational agent; (5) a history of prior radiotherapy.

Each patient underwent physical examination, endoscopy, chest CT, abdominal CT or MRI, and cervical ultrasound or CT at baseline to get an accurate clinical stage. Whole-body PET-CT was recommended but not required for all the patients.

### Ethical considerations

All patients provided written informed consent before enrollment in this study. Institutional ethics committee of Fudan University Shanghai cancer center approval was obtained before the study.

### Radiotherapy

External beam radiotherapy was administered at a total dose of 61.2 Gy in 34 fractions or 50.4 Gy in 28 fractions, with five fractions per week for 6 or 7 weeks. The prescribed dose mainly depended on the tumor volume and the limitations of the dosing of normal tissues. Radiation therapy was administered using intensity-modulated radiation therapy (IMRT) via a 6 MV X-ray beam. For patients with newly diagnosed esophageal SCC, the gross tumor volume (GTV) comprised the primary tumor and the involved lymph nodes (LNs) based on imaging, endoscopy and biopsy. Positive lymph nodes were defined as lymph nodes ≥1 cm along the shortest axis and ≥ 5 mm along the tracheoesophageal groove. The clinical target volume (CTV) of the esophageal tumor (CTVeso) was defined with a 3.0-cm cranial-caudal margin and a 0-cm margin in other directions. The CTVln was defined as equal to the GTVln. The planning target volume (PTV) included the CTVeso and CTVln with a 1-cm margin in all directions, except in situations where the spinal cord tolerance would have been exceeded. For patients with recurrent disease, the GTV included recurrent lesions defined by imaging, endoscopy and biopsy. PTV was defined as the GTV with an additional 1-cm margin in all directions.

The plan was optimized as follows: (1) 99% of the PTV was covered by 95% of the prescribed dose; (2) 95% of the PTV volume was covered by the prescribed dose; (3) the maximum dose did not exceed 110% of the prescribed dose based on a continuous volume of < 1 cm^3^ in the PTV; (4) the maximum dose of the PTV did not exceed 120% of the prescribed dose. For patients without prior RT, the normal tissue constraints for the critical organs were as follows: a maximum spinal cord point dose of ≤45 Gy; a percentage of the total lung volume receiving ≥20 Gy (lung V20) of ≤30% and a mean lung dose of ≤16 Gy; a mean heart dose of ≤30 Gy; a maximum gastrointestinal dose of ≤54 Gy.

### Chemotherapy

Concurrent chemotherapy was administered starting on the first day of radiotherapy and comprised 5 or 6 cycles of docetaxel (25 mg/m^2^) and cisplatin (25 mg/m^2^) on days 1, 8, 15, 22, 29 and 36. Thirty minutes before treatment with docetaxel, patients were premedicated with 10 mg of dexamethasone intramuscularly. Chemotherapy was delayed if the absolute granulocyte count was < 1.5 × 10^9^/L, the WBC count was < 3.0 × 10^9^/L, hemoglobin was < 80 g/L or the platelet count was < 75 × 10^9^/L. If a grade 4 hematological toxicity occurred, then chemotherapy was suspended until recovery, and the dose of docetaxel and cisplatin was subsequently reduced by 25%.

### Follow-up

Patients were evaluated via physical examination, chest and abdominal CT, and barium esophagography 4–6 weeks after CRT and then every 3 months for the first 2 years after treatment, every 6 months during the following 3 years, and yearly thereafter. Digestive endoscopy was performed if patients had symptoms of dysphagia or abnormal manifestations during barium esophagography.

### Toxicity

Adverse reactions to chemoradiotherapy were evaluated according to the Common Terminology Criteria for Adverse Events (CTCAE) version 4.03. Acute toxicities were defined as adverse effects that occurred between the start of treatment and 3 months after CRT. An adverse effect occurring more than 90 days after the completion of CRT was defined as late toxicity. Patients who received RT and at least one cycle of chemotherapy were included in safety analysis.

### Statistics

The primary endpoint of the present study was OS, and the secondary endpoints were progression-free survival (PFS), locoregional control and treatment-related toxicities. OS was calculated from the first day of CRT up until the time of the last follow-up or death. PFS was measured from the date of treatment initiation to the date of progression, death or last follow-up. Locoregional control was measured from the date of treatment initiation to the date of locoregional failure, which was defined as recurrence or persistence within the irradiated field. Recurrences outside the irradiated field were considered as distant metastasis. A sample size of 49 was required to accept the hypothesis that an increase in the median OS from 7 months to 16 months had occurred with a power of 80% and a standard error of 0.05. A 10% adjustment for dropouts resulted in a sample size of 54 patients. Survival data were analyzed according to the intention-to-treat population. Survival curves were estimated using the Kaplan-Meier method. The survival differences between the newly diagnosed group and the recurrent group were assessed with log-rank tests. A *p* value of less than 0.05 was considered significant. Statistical analyses were performed using SPSS statistical software (version 19.0).

## Results

**Fig. 1:**
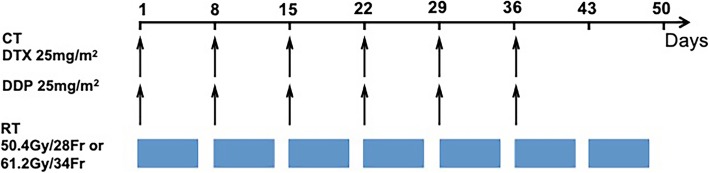
Treatment schedule of chemoradiotherapy with weekly docetaxel and cisplatin. DTX: docetaxel. DDP: cisplatin

From October 2015 to December 2016, 54 patients were enrolled in this study. Figure 1 shows the progression of the study phases. The baseline characteristics of patients are shown in Table 1. Of all patients, 36 (66.7%) were newly diagnosed with T4 or M1 LNM metastasis, and 18 (33.3%) had postoperative locoregional recurrences without prior neoadjuvant or adjuvant radiotherapy. The median interval between surgery and relapse was 15.5 months (range: 6–50 months). All patients were assessed with cervical, chest and abdominal imaging scan to identify primary and metastatic lesions.19 patients undertook a whole-body PET-CT scan before treatment.

**Table 1:** Baseline clinical characteristics (*n* = 54)

Variable		Value (%)
Median age (range)		60 (42–72)
Sex	Male	47 (87.0)
	Female	7 (13.0)
ECOG performance status	0–1	54 (100)
Smoking	non-smoker	20 (37.0)
	smoker	34 (63.0)
Drinking	No	21 (38.9)
	Yes	33 (61.1)
Main tumor location	cervical	1 (1.9)
	upper thoracic	11 (20.4)
	middle thoracic	25 (46.3)
	lower thoracic	15 (27.8)
	two sites	2 (3.8)
Histology	Squamous cell carcinoma	54 (100)
TNM stage (UICC 6th)	T4N0-1 M0(III)	14 (25.9)
	T1-4N0-1M1a(IVa)	7 (13.0)
	T1-4N0-1M1b(IVb)	15 (27.8)
	Recurrences	18 (33.3)

Abbreviations: *TNM* tumor, node, metastasis, *UICC* International Union Against Cancer

### Treatment compliance and toxicities

All 54 patients completed at least one cycle of concurrent chemotherapy and 41 of them (75.9%) completed all prescribed cycles. Only 18 patients (33.3%) received 1 or 2 extra cycles of consolidation chemotherapy. The consolidation chemotherapy consisted of docetaxel (60 mg/m^2^) plus cisplatin (25 mg/m^2^) for 3 days after CRT. Chemotherapy dose reduction was necessary in 9 patients due to grade 4 neutropenia. Twenty patients were given a reduced prescribed dose of 50.4 Gy in 28 fractions, and the rest were given 61.2 Gy in 34 fractions. For 18 patients with recurrence, the GTV included an anastomosis in 3 patients, the supraclavicular LNM in 3 patients, the mediastinal LNM in 10 patients, the left gastric lymphatic LNM in 1 patient and both the supraclavicular and mediastinal LNM in 2 patients. Fifty-one of 54 patients (94.4%) completed the entire course of radiation. Three patients terminated radiation ahead of schedule due to intolerance. Seven patients delayed CRT due to hematologic toxicities or weakness; nevertheless, concurrent chemotherapy delay and cessation in the remaining 6 cases were also mainly caused by treatment-induced toxicities. The median delay time was 6 days for chemotherapy (ranging from 3 to 14 days) and 3 days for RT (ranging from 1 to 14 days).

At baseline, 40 of 54 patients (74.1%) reported dysphagia. One patient received a gastrostomy feeding tube before treatment. After CRT, 35 patients (87.5%) reported the improvement or resolution of dysphasia. Three of 54 patients (7.5%) reported no change in dysphagia, and two patients (5.0%) reported the worsening of dysphagia. Both of the latter patients required esophageal dilatation for esophageal stricture after treatment.

Because all patients received at least one cycle of chemotherapy, the safety population in this study was equal to the intention-to-treat population. Treatment-related toxicities were evaluated based on CTCAE v4.03 and are listed in Table 2. Esophagitis was the most frequent acute toxicity, and in most cases, it was grade 1. Intravenous nutritional support was given as needed in 6 patients with grade 3 esophagitis. Forty-seven of 54 patients had hematologic toxicities, including anemia, neutropenia and thrombocytopenia. Severe hemocytopenia occurred in 16 patients with grade 3-4 neutropenia, two patients with grade 3 anemia and one patient with grade 3 thrombocytopenia. Most patients recovered with supportive care, including subcutaneous stimulating factor injection. Fatigue and anorexia were also commonly observed. Grade 3 radiation pneumonitis occurred in 3 patients, and only one of these patients was in the recurrent group. One patient from newly diagnosed group died from grade 5 radiation pneumonitis after CRT. One patient had a tracheo-esophageal fistula in the upper esophagus 2 months after the completion of CRT, and another patient had esophageal hemorrhage without clear evidence of progression (Grade 5 esophagitis) 1 month after CRT. Both patients were from recurrent group and died within a short period. Late toxicities were mild. Seven patients had grade 1 pleural effusion after CRT, but none of them needed therapeutic thoracentesis. Grade 1 cardiac disorder occurred in 14 patients, and all of them were diagnosed by electrocardiogram without clinical symptoms. In general, there was no significant difference between the newly diagnosed group and the recurrent group in the incidence of grade 3 or higher AE [23(63.9%) v 8(44.4%), respectively, *p* = 0.173]. Most acute and late toxicities were similar between the two groups except pulmonary fibrosis [13 (36.1%) vs 2 (11.1%), respectively, *p* = 0.012].

**Table 2:** Acute and late Treatment-related toxicity

Toxicity	Newly diagnosed group (*n* = 36)					Recurrent group (*n* = 18)				
	Grade 1	Grade 2	Grade 3	Grade 4	Grade 5	Grade 1	Grade 2	Grade 3	Grade 4	Grade 5
Haematological										
Leukocytopenia	7 (19.4)	11 (30.6)	4 (11.1)	7 (19.4)	0	4 (22.2)	5 (27.8)	3 (16.7)	2 (11.1)	0
Anaemia	12 (33.3)	8 (22.2)	1 (2.8)	0	0	8 (44.4)	4 (22.2)	1 (5.6)	0	0
Thrombocytopenia	7 (19.4)	4 (11.1)	0	0	0	1 (5.6)	1 (5.6)	1 (5.6)	0	0
Hypokalemia	1 (2.8)	0	5 (13.9)	0	0	1 (5.6))	0	1 (5.6)	0	0
Hyponatremia	3 (8.3)	0	2 (5.6)	0	0	1 (5.6)	0	0	0	0
Non-Hematological										
Fever	7 (19.4)	2 (5.6)	0	0	1 (2.8)	3 (16.7)	1 (5.6)	0	0	0
Esophagitis	21 (58.3)	3 (8.3)	4 (11.1)	0	0	9 (50.0)	1 (5.6)	2 (11.1)	0	1 (5.6)
Tracheo-oesophageal fistula	0	0	0	0	0	0	0	0	0	1 (5.6)
Pneumonitis	16 (44.4)	6 (16.7)	2 (5.6)	0	1 (2.8)	4 (22.2)	4 (22.2)	1 (5.6)	0	0
Fatigue	12 (33.3)	7 (19.4)	8 (22.2)	0	0	4 (22.2)	2 (11.1)	3 (16.7)	0	0
Nausea	12 (33.3)	3 (8.3)	0	0	0	1 (5.6)	2 (11.1)	0	0	0
Vomiting	5 (13.9)	1 (2.8)	0	0	0	1 (5.6)	1 (5.6)	0	0	0
Anorexia	11 (30.6)	4 (11.1)	5 (13.9)	0	0	4 (22.2)	2 (11.1)	2 (11.1)	0	0
Diarrhea	2 (5.6)	1 (2.8)	0	0	0	0	1 (5.6)	0	0	0
Dermatitis	6 (16.7)	2 (7.4)	1 (2.8)	0	0	0	2 (11.1)	3 (16.7)	0	0
Hiccups	1 (2.8)	0	0	0	0	1 (5.6)	0	0	0	0
Hoarseness	5 (13.9)	1 (2.8)	0	0	0	3 (16.7)	0	0	0	0
Arthralgia And Myalgia	2 (5.6)	0	0	0	0	0	0	0	0	0
Weight Loss	8 (22.2)	3 (8.3)	0	0	0	3 (16.7)	1 (5.6)	0	0	0
Pleural effusion	6 (16.7)	0	0	0	0	1 (5.6)	0	0	0	0
Cardiac disorder	10 (27.8)	0	0	0	0	4 (22.2)	0	0	0	0
Pulmonary fibrosis	13 (36.1)	0	0	0	0	2 (11.1)	0	0	0	0
Esophageal stenosis	2 (5.6)	0	0	0	0	0	0	0	0	0

### Response to treatment and patterns of failure

The radiological response to treatment was determined according to esophagoscopy findings and CT scans 4–6 weeks after treatment. The results were shown in Table 3. At the time of the latest analysis, 15 patients had locoregional progression within the irradiated field. Twenty-three patients had distant metastases, including 7 in the lungs, 2 in bones, 4 in liver, 2 in pleura, 2 in the adrenal gland, 2 in regional lymph nodes outside the irradiated field and 9 in non-regional lymph nodes.

**Table 3:** Radiological response to treatment at 4–6 weeks after the concurrent chemoradiotherpay

Response	Overall	III	IVa	IVb	Recurrences
CR	21 (38.9)	8 (14.8)	3 (5.6)	3 (5.6)	7 (13.0)
PR	21 (38.9)	5 (9.3)	3 (5.6)	7 (13.0)	6 (11.1)
SD	7 (13.0)	1 (1.9)	0	3 (5.6)	3 (5.6)
PD	3 (5.6)	0	0	1 (1.9)	2 (3.7)
Missing	2 (3.7)	0	1 (1.9)	1 (1.9)	0
Total	54 (100.0)	14 (25.9)	7 (13.0)	15 (27.8)	18 (33.3)

### Survival

**Fig. 2:**
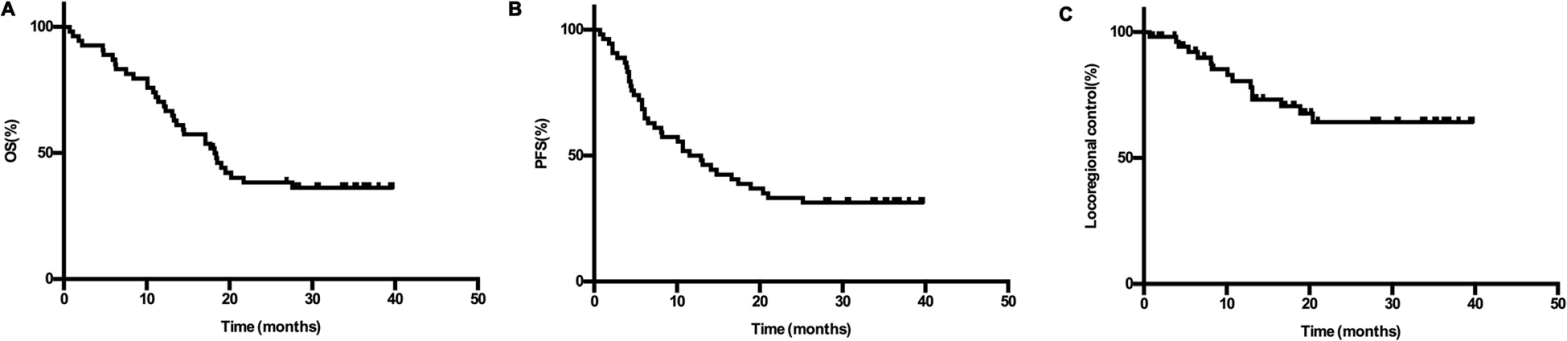
Overall survival (**a**), progression-free survival (**b**) and locoregional control (**c**) of the 54 patients. OS, overall survival; PFS, progression-free survival

After a median follow-up of 18.1 months (range:1.0–39.7 months) for the whole cohort, 34 patients had died, including 6 from locoregional tumor progression, 16 from distant metastases, 5 from both locoregional and distant metastases, 1 from septic shock during treatment, 1 from radiation pneumonia, 1 from infectious pneumonia, 1 from tracheoesophageal fistula, 1 from hemorrhage after radiation, 1 from severe weakness after CRT and 1 due to an unknown reason. The estimated median overall survival was 18.2 months (95% confidence interval [CI], 13.7–22.7 months), and the OS rate at 1 and 3 years was 70.4 and 36.4%, respectively. The 1-year and 3-year locoregional control rates were 80.6 and 64.3%, respectively. A median PFS time of 11.5 months (95% CI, 6.7–16.3 months) and 1- and 3-year PFS rates of 50.0 and 31.5% were observed (Fig. 2).

**Fig. 3:**
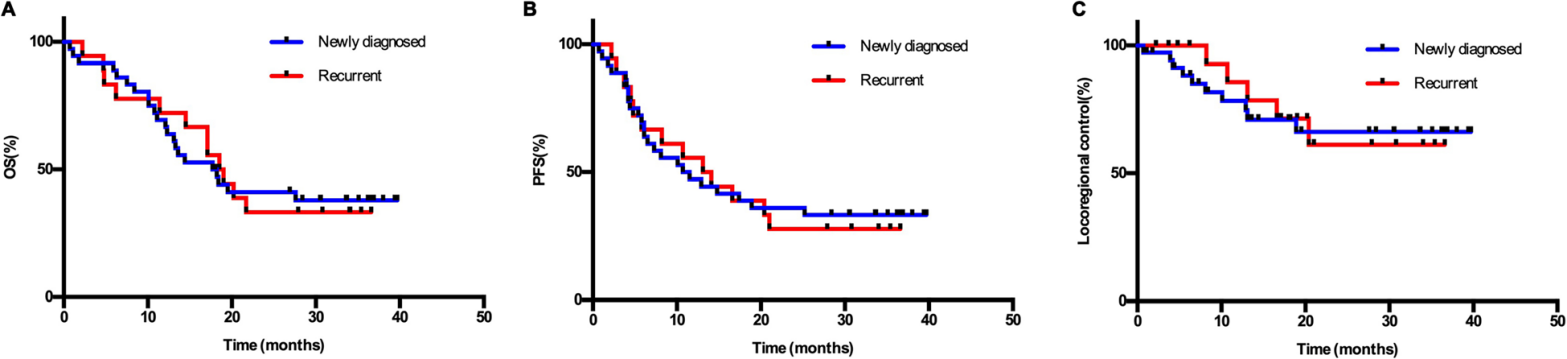
Overall survival (**a**), progression-free survival (**b**) and locoregional control (**c**) according to enrolled group (newly diagnosed vs. recurrent). OS, overall survival; PFS, progression-free survival

The median OS, locoregional control rate and PFS for the enrolled group (newly diagnosed vs. recurrent) are shown in Fig. 3. No significant survival, locoregional control or PFS differences at each stage were noted (*p* = 0.978, 0.857 and 0.910, respectively).

## Discussion

The systemic activity of docetaxel has been demonstrated either alone or in combination with other chemotherapeutic agents in advanced esophageal cancer [17, 20, 21]. In addition, docetaxel has also been used in chemoradiotherapy for esophageal cancer as a potent radiosensitizer [22, 23]. The response rate in these studies ranged from 47 to 89.5%. However, few patients were enrolled in most studies. Font et al. reported that weekly docetaxel treatment combined with 66 Gy-RT in 27 patients with inoperable esophageal cancer resulted in a median OS of 6 months, and 35 and 12% patients were still alive after 1 and 3 years with radiological response rates of 26% (complete) and 24% (partial) [23]. It seems that outcomes when using a combination of docetaxel with other chemotherapeutic agents is more efficient than when using docetaxel alone. Li et al. conducted a phase II study of docetaxel (60 mg/m2) and cisplatin (80 mg/m2) treatment every 3 weeks during the period of radiotherapy for advanced esophageal cancer, and the median OS was reported to be 22.6 months, the PFS rate and OS after 3 years were 29.2 and 36.7%, respectively [15]. A phase II study of 36 patients with advanced esophageal SCC (55.6% of patients had stage III tumors) who received 20 mg/m^2^ docetaxel and 25 mg/m^2^ cisplatin weekly with concurrent 54-Gy RT resulted in a median time to progression of 13.5 months, and the median OS was 26.9 months [24]. In the present study, we incorporated DP weekly concurrentwith radiotherapy to treat patients with T4 or M1 LNM esophageal SCC. Our results showed that the tumor response rate was 80.8%; the median OS and PFS time were 18.2 months and 11.5 months, and the 3-year OS and PFS were 36.4 and 31.5%, respectively. These results were more promising than previous similar studies of PF and TC-based CRT [9–11, 18]. However, without a prospective randomized comparison, we can’t conclude DP regimen is superior to than PF or TC regimen for locally advanced and recurrent esophageal SCC. Our PFS was similar to that observed in previous studies of advanced esophageal cancer which used DP regimen, but the median OS appeared to be worse. This might be due to the relatively worse stages and shorter follow-up periods of the enrolled patients in our study. However, the 3-year OS was similar to that found in patients treated concurrently with CRT and the DP regimen. We speculate that this might result from the high dose of radiation therapy that our patients received. However, these studies did not use a stable dose of drugs, and more data are needed for comparison. In another phase II study evaluating CRT with docetaxel, cisplatin and 5-fluorouracil treatment in 42 patients with untreated T4 tumors and/or M1 LNM, the median PFS was 11.1 months, and the median survival was 29.0 months with a survival rate of 43.9% after 3 years [24]. The addition of 5-fluorouracil caused the OS rate to slightly improve, but the occurrence of grade 3 or higher toxicities obviously increased. Our results also indicated that the median PFS and OS in the newly diagnosed group and the postoperative recurrence group were quite similar. Locoregional recurrence, especially lymph node recurrence (LNR), is the most frequent recurrence pattern found in esophageal SCC and comprises 27-45% of cases [25, 26]. The number of recurrent tumors has been considered an independent prognostic parameter after curative therapy in some studies [26, 27]. However, no optimal treatment strategy has yet been established for patients with locoregional recurrences. Makino et al. [28] and Shimada et al. [29] reported that patients with only one recurrent tumor (including both LN and organ metastasis) had a significantly better prognosis than those with multiple recurrent tumors. In Makino’s study, 3/4 of patients with solitary LNR eventually developed distant metastasis [29]. In patients with recurrent esophageal SCC treated with DP as a first-line therapy, the median PFS was 5.0 months, and the median survival was only 8.3 months. Therefore, we supposed that local treatment plus systemic chemotherapy might be a better treatment strategy for patients with unresectable locally recurrence, and encouraging results were found in our study when using this strategy.

We used the 6th edition AJCC staging system in this study because it was more practical for nonsurgical patients. However, in the 7th and 8th editions of the AJCC staging system for esophageal carcinoma, the definition of regional LN has changed, and the previous subclassifications M1a and M1b have been eliminated. This change means that the same treatment strategies could now be used for M1a and M1b nonvisceral LN metastasis according to the previous staging system. If LN metastases could be covered by a single radiation field and treated appropriately with a potent radiosensitizer, the survival of patients with M1a and M1b nonvisceral LN disease would be improved.

The ratio of local/regional failure and persistence of disease after 2 years in both the high-dose and standard-dose arms was more than 50% in RTOG 94–05 [5]. In the present study, the locoregional control rate was 64.3% after 3 years and seemed to be similar to the studies using TC [11, 30] but higher than that found in previous studies of PF. Furthermore, the initial failure pattern was so different that there were fewer patients with persistent tumors in our study. In previous studies, 33 to 37% of patients with esophageal cancer had tumor persistence after CRT [4–6], while the rate of stable and progressive diseases was only 18.6% in the present study. Considering the more advanced stages of our patients, it is encouraging to observe that docetaxel may be a better radiation sensitizer than conventional chemotherapy regimens for esophageal SCC. In a previous study that enrolled nine patients with advanced thoracic esophageal SCC who had a T4 tumor and/or distant M1 LNM, the response rate was 67%, and the median survival time was 16.2 months with a 2-year survival rate of 38.9% after treatment with weekly concurrent docetaxel and 60-Gy radiation treatment [19].

Our study shows that 21 of 54 patients died because of distant metastases. This may be because of the advanced stages of the enrolled patients. Therefore, the effect of docetaxel on distant metastases in patients at varying stages needs further study. However, a 3-year OS rate of 36.4% seems to be equivalent to that achieved with conventional regimens (3-year overall survival rate of approximately 30%) in Stages I to III^45^. As the control of locoregional disease is further improved, the prevention of distant metastases will need to be addressed in the future.

A previous phase I trial has showed that the maximum tolerated dose of docetaxel and cisplatin was 30 mg/m^2^ per week in esophageal SCC [31]*.* The dose of docetaxel and cisplatin in our study was rationally determined, and our results indicated that major treatment-related toxicity was related to hematologic toxicity and esophagitis. According to the experiences in using concurrent CRT with docetaxel in previous studies, differences in the schedules used for docetaxel treatment are likely the main reason for the different toxicities. Patients may have more severe hematologic toxicity due to docetaxel when treated at 3-week intervals than when treated weekly. In our study, 13 and 16.7% of patients had grade 3 and 4 neutropenia, respectively. In a group of patients treated with chemoradiotherapy with docetaxel and cisplatin for 3 weeks, the proportions of patients with grade 3 and 4 neutropenia were 33.9 and 20.3%, respectively [15]. In another similar study the incidences of grade 4 neutropenia, thrombocytopenia and anaemia were 57.8, 4.4 and 4.4% [32]. However, in a similar study using weekly docetaxel and cisplatin treatment with CRT, grade 3-4 hematologic toxicity was observed in less than 5% of patients [24]. Considering that we used higher doses of docetaxel and RT and that some of the patients with recurrence had received neoadjuvant or adjuvant chemotherapy before, we believe our results are reasonable and acceptable. Acute pulmonary toxicity of grade 3 and above occurred in 4 patients (7.4%), but one patient with newly diagnosed esophageal SCC died from radiation pneumonitis after treatment. All four of these patients received radiation at a dose of 61.2 Gy. The INT0123 study investigated whether a higher radiation dose combined with PF was able to achieve any improvement in survival [17]. However, RT doses higher than 60 Gy are often used in clinical practice. It needs to be confirmed that the use of a higher RT dose with IMRT will not cause increased therapeutic mortality and morbidity. Three other grade 5 toxicities were observed in this study, two of which were from the recurrent patients with large mass that invades adjacent tissues. Considering the small sample size of the study, we don’t have enough evidence that the recurrenct group is less safe than the newly dignosed group when receiving concurrent CRT. Late toxicities were less frequently observed in our study. Only seven patients (12.9%) experienced grade 1 pleural effusion after CRT in our study, while in a study by Li, a higher probability (18.6%) of pleural effusion was reported, and 6.8% of patients required therapeutic thoracentesis [15]. In our study the incidences of acute and late toxicities between newly diagnosed and recurrent patients were not significantly different except pulmonary fibrosis after CRT. We found pulmonary fibrosis was more common in newly diagnosed group, which may be caused by a larger radiation field for these patients than patients with locoreginal recurrences.

## Conclusion

CRT with concurrent weekly docetaxel and cisplatin treatment in patients with stage T4 or M1 LNM esophageal SCC was tolerable and effective. This treatment strategy was also a convenient and available regimen for patients with locoregional recurrence. Ultimately, clinical trials with larger sample sizes and comparisons with conventional PF regimens will be needed.

## Data Availability

Our data were collected from patients treated at the Department of Radiation Oncology, Fudan University Shanghai Cancer Center from October 2015 to December 2016. The datasets used and/or analyzed during the current study are available from the corresponding author on reasonable request.

## Abbreviations

SCC: Squamous cell carcinoma
LNM: Lymph node metastasis
CRT: Chemoradiotherapy
PF: Cisplatin with 5-fluorouracil
DP: Docetaxel and cisplatin
RT: Radiotherapy
TC: Paclitaxel and carboplatin
3DCRT: 3-dimensional conformal radiation therapy
IMRT: Intensity-modulated radiation therapy
OS: Overall survival
PFS: Progression-free survival
GTV: Gross tumour volume
CTV: Clinical target volume
PTV: Planning target volume
UICC: International Union Against Cancer
CI: Confidence interval
CTCAE: Common Terminology Criteria for Adverse Events
LN: Lymph node
LNR: Lymph node recurrence
ECOG: Eastern cooperative oncology group
